# Supplementary material for: On-chip silicon electro-optical modulator with ultra-high extinction ratio for fiber-optic distributed acoustic sensing
Source: Nat Commun. 2023 Nov 16;14:7409. doi: 10.1038/s41467-023-43244-9 (PMC10654388; doi:10.1038/s41467-023-43244-9)
Supplement: Supplementary file 1 — Supplementary Information [file 41467_2023_43244_MOESM1_ESM.pdf]

Supplementary Information

On-chip silicon electro-optical modulator with ultra-high extinction ratio for fiber-optic distributed acoustic sensing

Zhuo Cheng[1,6], Xiaoqian Shu[1,6], Lingmei Ma[1,6], Bigeng Chen[1*], Caiyun Li[1], Chunlei Sun[2], Maoliang Wei[3], Shaoliang Yu[4], Lan Li[2], Hongtao lin[3], Yunjiang Rao[1,5*]

[1]Research Center for Optical Fiber Sensing, Zhejiang Laboratory, Hangzhou 311100, China
[2]Key Laboratory of 3D Micro/Nano Fabrication and Characterization of Zhejiang Province, School of Engineering, Westlake University, Hangzhou 310024, China
[3]College of Information Science and Electronic Engineering, Zhejiang University, Hangzhou 310027, China
[4]Research Center for Intelligent Optoelectronic Computing, Zhejiang Laboratory, Hangzhou 311100, China
[5]Key Laboratory of Optical Fiber Sensing and Communications (Education Ministry of China), University of Electronic Science and Technology of China, Chengdu 611731, China
[6]These authors contributed equally: Zhuo Cheng, Xiaoqian Shu, Lingmei Ma.
*chenbg@zhejianglab.com
*yjrao@uestc.edu.cn

Supplementary Note 1: Synthesis of a high-order coupled microring bandpass filter

Design of a high-order coupled microring bandpass filter and calculation of the coupling efficiencies between the microrings and input/output waveguides are performed based on a synthesis principle in analogous to the high-order electronic filter design.

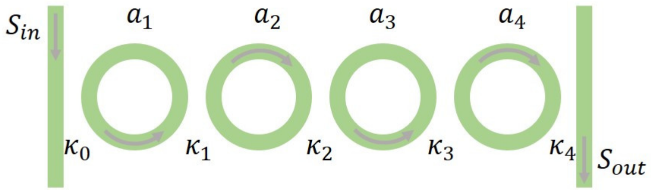

Supplementary Fig. 1. Schematic of a four serially-coupled microring filter.

As shown in Supplementary Fig. 1, the four identical ring resonators are serially coupled with input and output waveguides on the left and right, respectively. At steady state, the coupled-mode equations for the structure can be expressed as[1]:

$$A\boldsymbol{a} \equiv \begin{bmatrix} s+\dfrac{1}{\tau_1} & i\kappa_1 & 0 & 0 \\ i\kappa_1 & s & i\kappa_2 & 0 \\ 0 & i\kappa_2 & s & i\kappa_3 \\ 0 & 0 & i\kappa_3 & s+\dfrac{1}{\tau_2} \end{bmatrix} \begin{bmatrix} a_1 \\ a_2 \\ a_3 \\ a_4 \end{bmatrix} = \begin{bmatrix} -i\kappa_0 S_{in} \\ 0 \\ 0 \\ 0 \end{bmatrix} \tag{1}$$

where a_i (i=1, 2, 3, 4) is the time-varying electric field amplitude, κ_i (i=0, 1, 2, 3, 4) is the coupling coefficient in time domain for each coupling region, $1/\tau_1$ and $1/\tau_2$ are the external loss coefficients for coupling into the input/output waveguides, $s = i\Delta\omega$ ($\Delta\omega$ is detuning frequency), S_{in} is the input fields, respectively. Internal loss is considered negligible here. Therefore, the

electric field transmission of the coupled microring structure can be derived as

$$T(\mathrm{s}) = \frac{\kappa_0\kappa_1\kappa_2\kappa_3\kappa_4}{s^4 + s^3\left(\frac{1}{\tau_1}+\frac{1}{\tau_2}\right) + s^2\left((\kappa_1^2+\kappa_2^2+\kappa_3^2)+\frac{1}{\tau_1\tau_2}\right) + s\left(\frac{1}{\tau_2}(\kappa_1^2+\kappa_2^2)+\frac{1}{\tau_1}(\kappa_2^2+\kappa_3^2)\right) + \kappa_1^2\kappa_3^2 + \frac{\kappa_2^2}{\tau_1\tau_2}} \quad (2)$$

Using the concept of electronic filter design, $T(\mathrm{s})$ presents the form of an all-pole function and thus the serially-coupled microring structure can be regarded as an all-pole filter. Here we choose the classical Butterworth filter to design our coupled microring response for maximum passband flatness. The transfer function for a four order Butterworth filter is[2]

$$T(\mathrm{s}) = \frac{\kappa}{s^4 + 2.6131s^3 + 3.4142s^2 + 2.6131s + 1} \quad (3)$$

Note that the coupling coefficients of the high-order microring filter are symmetric distributed[3]. Then, by comparing the denominators of Supplementary Equation (2) and Supplementary Equation (3), the coupling coefficients $(\kappa_1, \kappa_2, \kappa_3)$ and loss coefficients $(1/\tau_1, 1/\tau_2)$ can be obtained. Furthermore, the coupling coefficients κ_0 and κ_4 can be calculated by[4]

$$\kappa_0 = \sqrt{2/\tau_1},\ \kappa_4 = \sqrt{2/\tau_2}, \quad (4)$$

Then the static coupling coefficient of optical field can be obtained by [1]

$$K_i = \sin(\frac{B\kappa_i}{FSR}) \text{ (for coupling between rings)} \quad (5)$$

$$K_i = \sqrt{\frac{2K}{1+K}} \text{ (for coupling between rings and bus waveguides)} \quad (6)$$

where FSR is the free spectral range of the microring resonator and B is the bandwidth parameter ($B = 2\pi FSR\sigma$). A desired filter bandwidth can be obtained by adjusting coefficient σ.

Supplementary Table 1 gives the calculated power coupling coefficients of a four-order coupled microring filter for different σ. Then the transmission spectrum can be obtained by using the transfer matrix method[5], as shown in Supplementary Fig. 2a. It can be found that stronger coupling leads to to larger bandwidth and smaller extinction ratio (ER). Because the coupling strength is determined by the physical gaps between ring resonators, which is limited the minimum linewidth of fabrication process, the bandwidth should be chosen carefully. Such a design process can be extended to filters with different orders. Supplementary Fig. 2b also given the transmission spectrum of different order filters with $\sigma = 0.02$.

Supplementary Table 1. Calculated power coupling coefficients of a four order serially-coupled microring filter

	waveguide and ring 1	ring 1 and ring 2	ring 2 and ring 3	ring 3 and ring 4	ring 4 and waveguide
$\sigma = 0.005$	0.07989	0.0007	0.0003	0.0007	0.07989
$\sigma = 0.01$	0.152	0.003	0.001	0.003	0.152
$\sigma = 0.019$	0.269	0.010	0.004	0.010	0.269
$\sigma = 0.05$	0.571	0.068	0.029	0.068	0.571

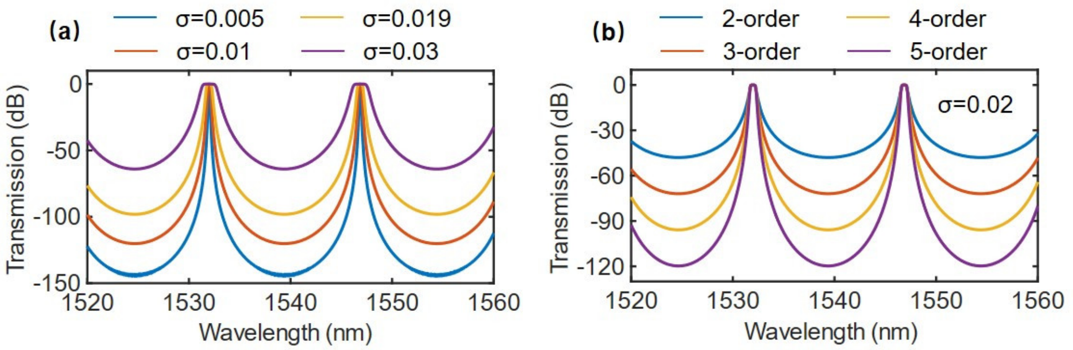

Supplementary Fig. 2. Calculated transmission spectra of high-order coupled microring filters. **a** Transmission spectra of four-order microring filters with different σ. **b** Transmission spectra of microring filters at $\sigma = 0.02$ with different orders.

Electro-optical modulators (EOMs) using different orders of coupled microring filters are designed and fabricated, as shown in Supplementary Fig. 3a. Considering the impact of fabrication errors on the device performance, the gap variations between the coupled rings as well as rings and bus waveguides are swept from -15 nm to 15 nm with respect to optimized values. The transmissions of the fabricated EOMs normalized to reference waveguides are presented in Supplementary Fig. 3b. It is obvious that higher order brings larger band roll-off. Besides, we can see the performance deviations are relatively less for the two- to four-order coupled rings in terms of out-of-band rejection and insertion loss, while the five-order coupled rings seem more sensitive to gap sizes. The sensitivity can be attributed to longer circulating optical path for the higher order structures, in which more phase error is accumulated.

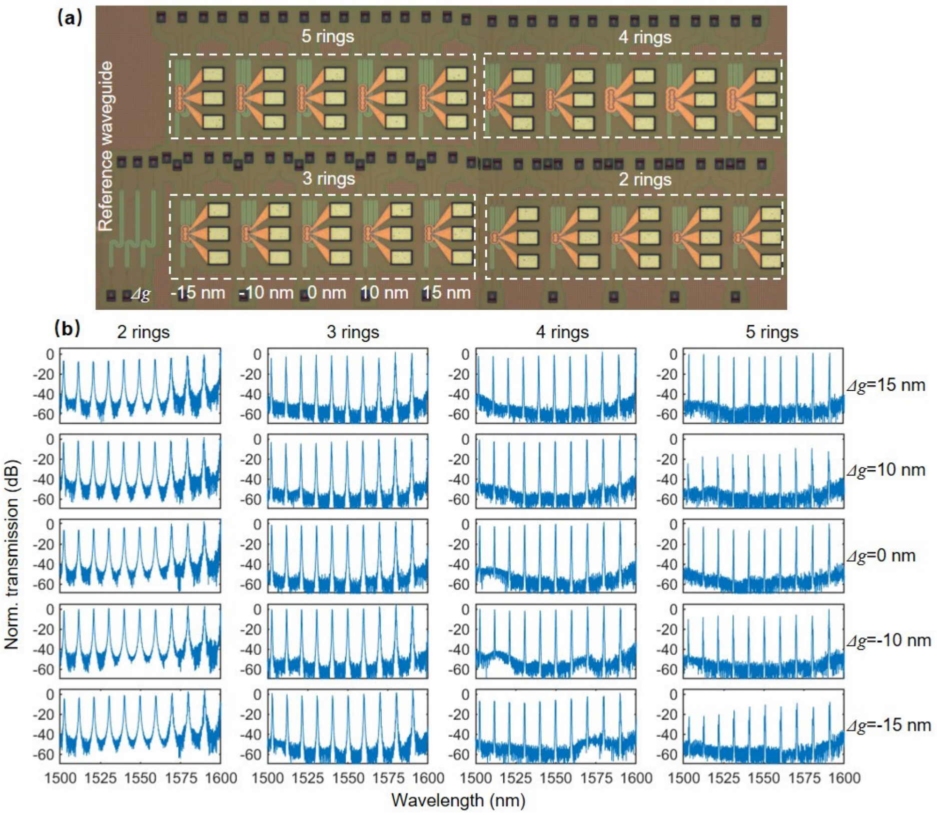

Supplementary Fig. 3. Measured transmissions of fabricated electro-optical modulators with order and gap variations. **a** Photograph of the fabricated electro-optical modulators (EOMs) with different orders of coupled microrings. **b** Normalized transmissions of the EOMs with different orders and gap sizes.

Supplementary Note 2: Electric-optical (EO) bandwidth of coupled microring EOM

Small-signal EO bandwidth of the EOM is characterized with a vector network analyzer (Keysight E5080B) and a 12-GHz impedance-matched photodiode. The input signal is applied to the GSG electrode via an RF probe (GGB 40A) and the modulated optical output is collected by the photodiode. Using the vector network analyzer, S21 signal is obtained as the EO response (see Supplementary Fig. 4). As a result, the 3-dB EO bandwidth of the EOM is about 0.72 GHz.

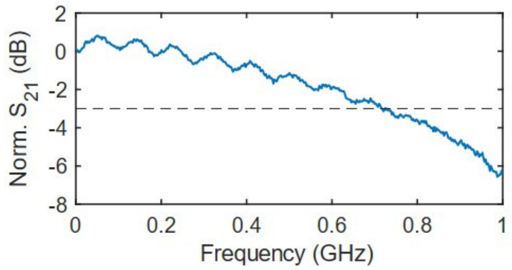

Supplementary Fig. 4. Measured electric-optical (EO) response of the fabricated coupled microring EOM.

Supplementary Note 3: Self-heterodyne measurement for ultra-high ER characterization

As mentioned in the main text, ultra-high ER of the generated optical pulses are characterized by self-heterodyne measurement with a large dynamic range. In the measurement (see Supplementary Fig. 5), the input light with a field amplitude E_i is split into two paths equally. One path is used as the reference (E_r) and the other path is used as signal light (E_s). The signal light is firstly modulated by the on-chip EOM and then passes through an acousto-optical frequency shifter (AFS) to shift the optical frequency by 200 MHz. Here, the fields of pulse and background CW light are denoted as E_{s_h} and E_{s_l}, respectively. Finally, the pulsed signal light is interfered with the CW reference light. The combined output is collected by a photodetector (PD) and received by an electrical spectrum analyzer (ESA).

Assuming $E_i = Ae^{i(\omega_0 t+\varphi_0)}$, we can have $E_r = Be^{i(\omega_0 t+\varphi_1)}$, $E_{s\_h} = C_1 e^{i((\omega_0+\Delta\omega)t+\varphi_2)}$ and $E_{s\_l} = C_2 e^{i((\omega_0+\omega_1)t+\varphi_3)}$ where ω_0 is the fundamental frequency and $\Delta\omega$ is the angular frequency shift (2π × 200 MHz). Hence, the ER of the EOM can be expressed as

$$\mathrm{ER} = \frac{|E_{s\_h}|^2}{|E_{s\_l}|^2} = (\frac{C_1}{C_2})^2 \tag{7}$$

After interference, the optical power of the combined signal become

$$\begin{aligned} I_{b\_h} &= \left(Be^{i(\omega_0 t+\varphi_1)} + C_1 e^{i((\omega_0+\Delta\omega)t+\varphi_2)}\right)\left(Be^{-i(\omega_0 t+\varphi_1)} + C_1 e^{-i((\omega_0+\Delta\omega)t+\varphi_2)}\right) \\ &= B^2 + C_1^2 + 2\mathrm{B}C_1\cos(\Delta\omega t + \varphi_1 - \varphi_2) \\ I_{b\_l} &= \left(Be^{i(\omega_0 t+\varphi_1)} + C_2 e^{i((\omega_0+\Delta\omega)t+\varphi_3)}\right)\left(Be^{-i(\omega_0 t+\varphi_1)} + C_3 e^{-i((\omega_0+\Delta\omega)t+\varphi_3)}\right) \\ &= B^2 + C_2^2 + 2\mathrm{B}C_2\cos(\Delta\omega t + \varphi_1 - \varphi_3) \end{aligned} \tag{8}$$

for the pulse and background, respectively. As a result, the AC component is on the 200-MHz carrier only. Using the ESA, we measure the electrical signal in power, which is proportional to the square of PD output voltage. At the meantime, the PD output is proportional to the interfered optical power. Therefore, the measured power at 200 MHz in ESA is linearly dependent on the signal light power in theory. Using the zero-span mode at 200 MHz, signal waveforms reflecting the optical pulses can be observed in time domain and the ER can be measured accordingly.

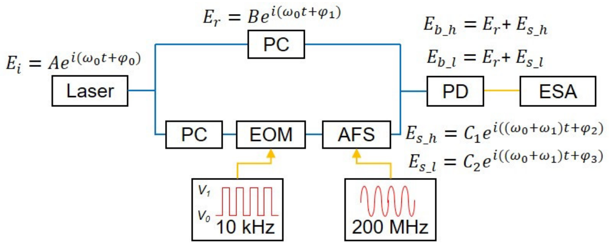

Supplementary Fig. 5. Schematic of the self-heterodyne measurement. PC: polarization controller, EOM: electro-optical modulator, AFS: acousto-optical frequency shifter, PD: photodetector, ESA: electrical spectrum analyzer.

To verify the accuracy of the self-heterodyne measurement, a calibration experiment is implemented by comparing the optical power of a CW signal light with the RF power received by the ESA. As shown in Supplementary Fig. 6a, a variable optical attenuator (VOA) is added into the signal path and 50% of its output is sent to the optical power meter (Santec MPM-210) while the rest is frequency-shifted for heterodyne measurement. The optical power of the reference stays constant all the time in both EOM measurement and the calibration. In this way, the received RF power dependent on optical power are obtained as given in Supplementary Fig. 6b, where a good linearity can be seen within the measurement range. The result at optical power of about -90 dBm slightly deviates from the linear trend because the RF power is approaching the noise floor of the ESA. For such a low optical power that is beyond the detection limit of our power meter, we cascade one more VOA with a known attenuation to measure the corresponding the RF beat power. According to the experiment and theory above, we believe that the measured RF power contrast from the pulse waveform in the ESA does reflect the actual ER of the modulated optical pulses.

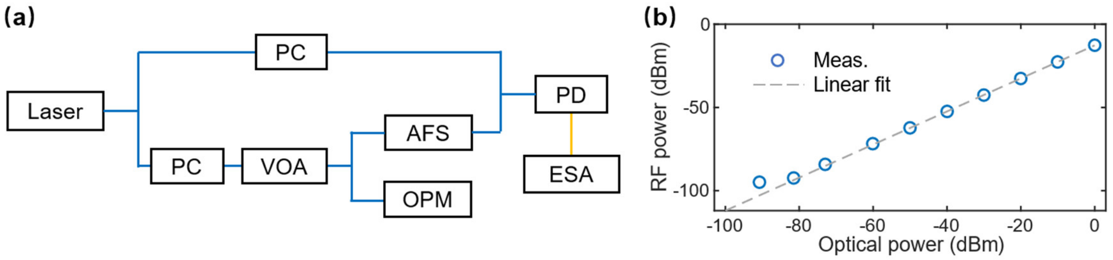

Supplementary Fig. 6. Calibration experiment of the self-heterodyne measurement. a Schematic of the experiment setup. PC: polarization controller, VOA: variable optical attenuator, AFS: acousto-optical frequency shifter, OPM: optical power meter, PD: photodetector, ESA: electrical spectrum analyzer. **b** Dependence of measured RF power by the ESA against optical power.

Supplementary Note 4: DAS system simulation

The simulation is performed using a model[6] as shown in Supplementary Fig. 7a to analyze the influence of ER on spatial crosstalk and noise floor numerically. The system parameters (peak power, duration, repletion, fiber length, etc.) are the same as those used in the experiment. The Rayleigh scattered signal (RBS) that enters the IMI is generated by the BefGen block using information provided by the NLL, PulseGen, ASE and FibGen blocks. To emulate the process of RBS generation, weak reflecting points with random reflectance are introduced at random positions along the fiber in BefGen. The RBS, which is the result of coherent superposition of the back-travelling pulses generated by the reflection points, displays a jagged appearance due to a combination of random destructive and constructive interference[7]. The IMI block then simulates the interference of the self-delayed RBS, producing three signals in 120° phase difference. Then, the detected signals from three photodetectors shown in Supplementary Fig. 7b are used to demodulate the phase that is

linearly dependent on the vibration applied to the fiber. The pulse ER can be adjusted in the PulseGen block. For each ER value, multiple frames of signal are generated and the phase signal is demodulated accordingly. Supplementary Fig. 7c-e show the demodulated phase, power spectrum density and crosstalk for ER of 30 dB and 70 dB, respectively, for clear comparison. Significant differences in crosstalk can be seen between the different ERs (Supplementary Fig. 7d and 7e), which agree well with our experiment. By changing the ER and repeating the simulation, spatial crosstalk and noise floor along the whole fiber against ER are both obtained as summarized by Figs. 5c and 5f in main text, respectively.

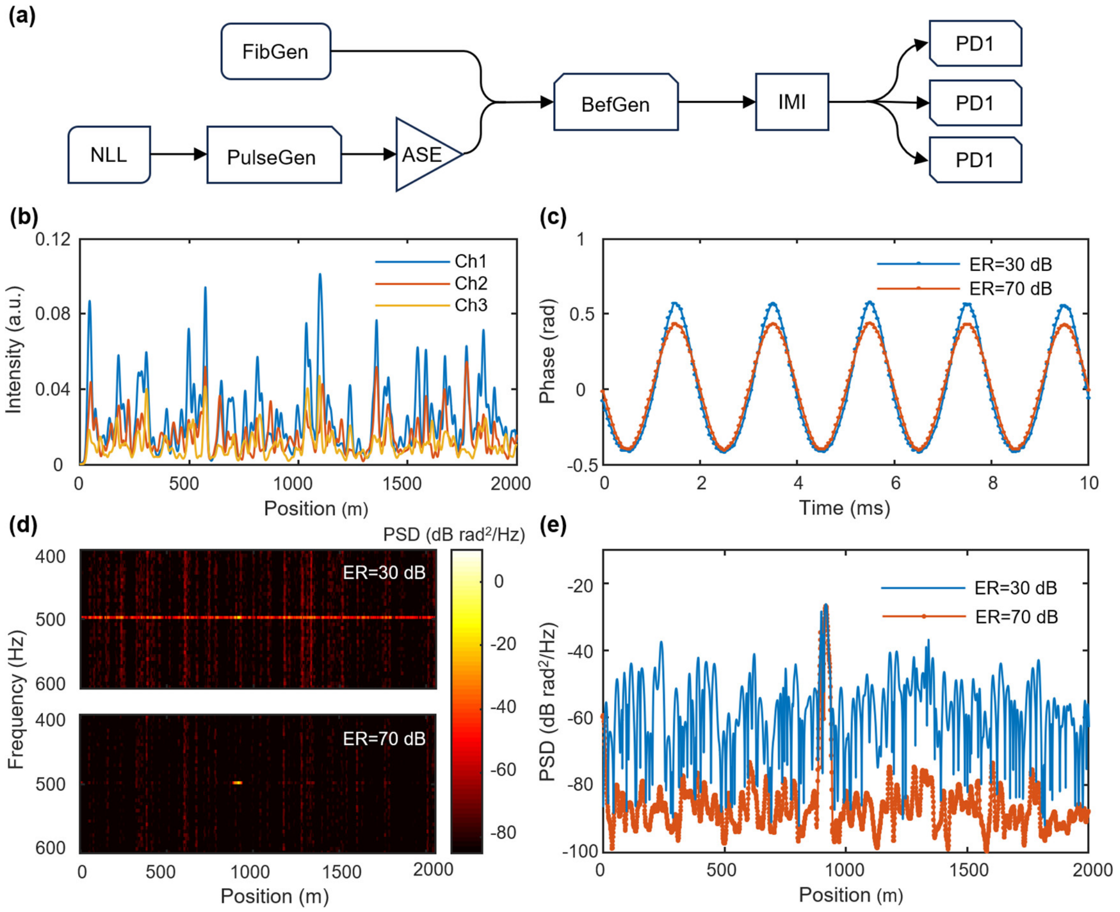

Supplementary Fig. 7. Simulation of the distributed acoustic sensing system. a Flowchart of the numerical model used to simulate the operation of phase-sensitive optical time domain reflectometer (Φ-OTDR). NLL: narrow-linewidth laser, PulseGen: probe pulse generation, FibGen: fiber generation, ASE: amplified spontaneous emission, BefGen: backscattering electric field generation, IMI: imbalanced Michelson interferometer, PD: photodetector. **b** The outputs of PD1, PD2 and PD3 dependent on position in the OTDR scheme; **c-e** Demodulated phases, power spectrum density (PSD) as functions of position and crosstalk around signal frequency (500 Hz) for extinction ratios (ERs) of 30 dB and 70 dB.

Supplementary Note 5: Thermal tuning power analysis

Silicon has a relatively higher thermo-optic coefficient (*dn/dT*=1.86×10-4/K @300K) than a number of other integrated photonic materials (e.g., SiN and LNOI). Hence, thermal tuning with metallic heaters for silicon photonic circuit is usually more efficient. A previous study had shown an ultralow tuning power of 1.2 mW for π phase shift (2.4 mW per FSR) in silicon racetrack resonators with air trenches[8]. We also simulate the thermal heating of a 100-µm-long TiN heater on a silicon rib waveguide without air trenches. Supplementary Fig. 8a shows the simulated

temperature distribution in the cross-section with 50-mW heating power applied to the TiN strip. With the knowledge of thermo-optic coefficient of silicon[9], the induced phase shift of the rib waveguide against heating power is obtained as given in Supplementary Fig. 8b. As a result, the power for π phase shift in this case is as large as 16 mW owing to the thermal conduction with the surrounding solid. Therefore, we estimate that the power consumption would increase by 4.8 mW in maximum if heaters are incorporated above the four rings with air trenches around. In practice, the increment would be less as the ring resonance drift is usually much smaller than half of an FSR. The relatively short perimeter restricts the accumulated phase errors along the ring induced by fabrication errors. Summing up the heating power, the total power dissipation of the coupled-ring EOM is less than 10 mW, still in a substantial contrast to that (Watt-scale) of an AOM.

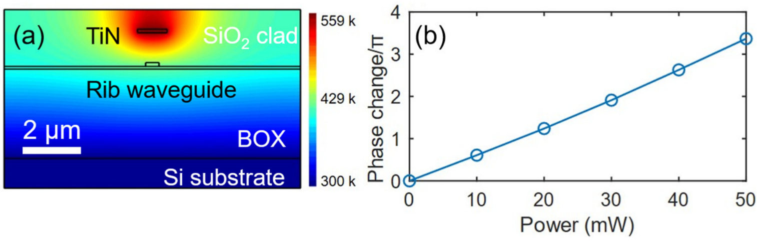

Supplementary Fig. 8. Thermal tuning power analysis. a Simulated temperature distribution in the cross-section with 50-mW heating power applied to the TiN strip. **b** Simulated phase shift of the rib waveguide dependent on heating power.

Supplementary References

1 Liu, H. & Amnon Y. Synthesis of high-order bandpass filters based on coupled-resonator optical waveguides (CROWs). *Opt. Express* **19** 17653-17668 (2011).

2 Marc T. Intuitive analog circuit design. *Elsevier* 2006.

3 Liu, D., He, J., Xiang, Y., Xu, Y. & Dai, D. High-performance silicon photonic filters based on all-passive tenth-order adiabatic elliptical-microrings. *APL Photonics* **7** 051303 (2022).

4 Little, B. E., Chu, S. T., Haus, H. A., Foresi, J. & Laine, J. -P. Microring resonator channel dropping filters. *J. Lightwave Technol.* **15** 998-1005 (1997).

5 Poon, J. K. S., Scheuer, J., Mookherjea, S., Paloczi, G. T., Huang, Y. &Yariv, A. Matrix analysis of microring coupled-resonator optical waveguides. *Opt. Express* **12** 90-103 (2004).

6 Li, C., Ma, L., Zhuang, Y., Long, J., Hu, W., Wang, J., Yan G. and Rao, Y. A High-Performance DAS System using Point-Backscattering-Enhanced Fiber and Study of its Noise Characteristics. *IEEE Sens. J.* Preprint at 10.1109/JSEN.2023.3324741. (2023)

7 Healey, P. Fading in heterodyne OTDR. *Electronics Letters* **1**, 30–32 (1984).

8 Po, D. et al. Thermally tunable silicon racetrack resonators with ultralow tuning power. *Opt. Express* **18** 20298-20304 (2010).

9 Cocorullo, G., Della Corte, F. G., & Rendina, I. Temperature dependence of the thermo-optic coefficient in crystalline silicon between room temperature and 550 K at the wavelength of 1523 nm. *Appl. Physics Letters* **74** 3338-3340 (1999).
